# Perspectives on faculty development: aiming for 6/6 by 2020

**DOI:** 10.1007/s40037-012-0006-3

**Published:** 2012-02-10

**Authors:** Yvonne Steinert

**Affiliations:** Centre for Medical Education, Faculty of Medicine, McGill University, Lady Meredith House, 1110 Pine Ave. West, Montreal, QC H3A 1A3 Canada

**Keywords:** Faculty development, Medical education

## Abstract

Faculty development has a key role to play in individual and organizational development. This perspective on faculty development, which builds on the *2020 Vision of Faculty Development Across the Medical Education Continuum Conference* and the *First International Conference on Faculty Development in the Health Professions,* describes six recommendations that we should consider as the field of faculty development moves forward: grounding faculty development in a theoretical framework; broadening the focus of faculty development to address the various roles that clinicians and basic scientists play; recognizing the role that faculty development can play in promoting curricular and organizational change; expanding our notion of how faculty members *develop* and moving beyond formal, structured activities to incorporate notions of work-based learning and communities of practice; making faculty development an expectation for all faculty members; and promoting scholarship in faculty development to ensure that research informs practice. Looking ahead, we should also consider strategies for leading change, collaborate across institutions and international borders, and work together to share lessons learned in research and practice.

Two recent conferences addressed important issues related to the future of faculty development. The first, entitled the *2020 Vision of Faculty Development Across the Medical Education Continuum Conference* [1], focused on core teaching competencies and barriers to effective teaching; relationship-centred care and the hidden curriculum that faculty members encounter; instructional technologies and biomedical informatics; lessons learned from continuing medical education; and research on faculty development [2]. The second, entitled the *First International Conference on Faculty Development in the Health Professions*, was held in May 2011 and welcomed over 300 participants from 28 countries to Toronto. The goal of this conference was to bring together international leaders and educators in faculty development, share best practices and research findings in this emerging field, and stimulate programme development, innovation and scholarship.

The goal of this article is to build on the theme of this journal and share perspectives on faculty development, based on the *2020 Vision of Faculty Development* *Across the Medical Education Continuum Conference* and lessons learned from the *First International Conference on Faculty Development in the Health Professions*. In doing so, I would like to highlight six recommendations for practice and research that we should consider as the field of faculty development moves forward: (1) grounding faculty development in a theoretical framework; (2) broadening the focus of faculty development to address the various roles that clinicians and basic scientists play; (3) recognizing the role that faculty development can play in promoting curricular and organizational change; (4) expanding our notion of how faculty members *develop* and moving beyond formal, structured activities to incorporate notions of work-based learning and communities of practice; (5) making faculty development an expectation for all faculty members; and (6) promoting scholarship in faculty development to ensure that research informs practice.

## The scope and definition of faculty development

The call for proposals for the *First International Conference on Faculty Development* *in the Health Professions* defined faculty development as ‘that broad range of activities that institutions use to renew or assist faculty in their roles’ [3]. It also re-affirmed the importance of using these activities to ‘assist faculty in their roles as teachers, educators, administrators, leaders and/or researchers’ [4]. Although this may be one of the more comprehensive definitions of faculty development to date, conference deliberations highlighted the diversity of terms used to describe this aspect of professional development as well as the fact that some languages have no equivalent. At the same time, the meaning of faculty development across cultures was revealing. For example, the Dutch term, *docentprofessionalisering,* loosely translates as the professionalization of teaching. This emphasis on professionalization, of both teachers and teaching, is intriguing and clearly aligns with a current focus on standards for teaching [5, 6]. The term is limited, however, in its emphasis on teaching (at the exclusion of other important faculty roles and tasks). In some ways, the French term, *formation professorale*, is more inclusive, as it is not restricted to teaching and refers to the ‘formation’ of the professorial role; the German term, *Personal*- *und Organisationsentwicklung*, is also of interest, as it emphasizes both individual and organizational development, another critical component of faculty development. Irrespective of the nomenclature, however, faculty development should encompass the skills relevant to the individual’s institutional and faculty position, and help to sustain their vitality, both now and in the future [7]. We should also consider Webster-Wright’s shift [8] from professional development to ‘continual professional learning’, which in many ways describes the ultimate goal of faculty development, as long as we remember that the word *faculty* is meant to be inclusive, referring to all individuals who are involved in the teaching and supervision of students in the health professions, at all levels of the continuum, in a wide range of contexts (e.g., in the classroom, at the bedside, in the outpatient clinic) and settings (e.g., the university, the hospital and the community).

## Grounding faculty development in a theoretical framework

MacDougall and Drummond [9] have observed that there is no clear theoretical framework to describe how medical teachers and educators develop. Theory is also noticeably absent from the faculty development literature [7]. And yet, a number of educational theories can be applied to faculty development and the development of faculty members, including constructivism [10], social learning [11], and self-efficacy [12]. However, in my opinion, situated learning [13] appears to be one of the most useful theoretical frameworks, as it is based upon the notion that knowledge is *contextually situated* and fundamentally influenced by the *activity, context*, and *culture* in which it is used [13]. This view of knowledge, as situated in authentic contexts, holds important implications for our understanding of how faculty members develop, as do the individual components of situated learning: cognitive apprenticeship (i.e., modelling, scaffolding, fading, and coaching), collaborative learning, reflection, practice, and articulation of learning skills [14]. In fact, reflection—and its role in faculty development—will warrant more attention in the future, for reflection ‘allows for the integration of theoretical concepts into practice, increased learning through experience, and enhanced critical thinking in complex situations’ [15]. Principles of adult learning [16] and experiential learning [17] are also pertinent in the design and delivery of faculty development programmes.

Closely tied to the notion of situated learning is the concept of ‘legitimate peripheral participation’ [18]. This social practice, which combines experiential learning and apprenticeship into a single theoretical perspective [19], is the process by which a novice becomes an expert. From this perspective, learners build new knowledge and understanding through gradual participation in the community of which they are becoming a part. As learners, they begin at the edge—or periphery—of the community, where because of their status as learners, they have what is called ‘legitimate peripheral participation,’ and through participation, they slowly adopt the knowledge, attitudes and behaviours of the community [20]. In many ways, faculty members go through this process as they take on their roles as teachers and educators. According to Wenger [21], social participation within the community is the key to informal learning; it is also a central ingredient in faculty development.

## Broadening the focus of faculty development

Both the faculty development literature and the conference proceedings focus predominantly on teaching and instructional effectiveness [22]; however, there is a critical need for faculty development activities to address the other roles of faculty members, including that of leader/administrator and researcher/scholar [7]. Faculty members’ own career development should also not be forgotten.

### A focus on leadership

Health care delivery, clinical practice, and medical education are all in a state of flux [2]. As a result, faculty members need to be prepared to deal with the rapid changes and shifting paradigms that are occurring in all three domains. Although some faculty development programmes have targeted leadership skills for health care professionals by focusing on skill acquisition [23], personal awareness [24], and increased knowledge of leadership style and organizational contexts [25], this area of professional development requires greater attention. In fact, faculty development initiatives should systematically address a wide range of topics, including personal and interpersonal effectiveness, leadership styles and change management, conflict resolution and negotiation, team building and collaboration, and organizational change and development [2]. Moving forward, we will need to develop leaders who can identify opportunities for change, respond effectively to emerging needs, and be prepared to take action.

### A focus on scholarship

Although research capacity building was an important component of faculty development in the 1990s [26, 27], much less has been written about the role of faculty development in promoting research and scholarship in this millennium. On the one hand, this may be due to a greater emphasis on advanced training in medical education [28, 29]; on the other hand, this observation may indicate the need to re-focus some of our energy towards research and scholarship. Boyer [30] has identified four categories of scholarship. The *scholarship of discovery* is synonymous with research in the traditional sense. The scholarship of *integration* has been defined as ‘making connections across the disciplines… illuminating data in a revealing way,’ whereas the scholarship of *application* has been likened to ‘service’ in one’s own field of knowledge, the application of theory into practice [30]. The *scholarship of teaching* is made possible through discovery, application or integration, and involves the capacity to effectively communicate one’s own knowledge, skills and beliefs. It has also been said that teaching becomes scholarship when it is made public, is available for peer review and critique, and can be reproduced and built on by other scholars [31]. Although many will agree that the promotion of scholarship—and helping educators to foster scholarly activities among colleagues—is an important aspect of faculty development, this component is often neglected. Moving forward, faculty development programmes could focus on definitions of scholarship, ways of promoting scholarship among colleagues and peers, methods of disseminating scholarly work, and ‘moving from innovation to scholarship’ [2]. A more traditional focus on research methods, grantsmanship and writing for publication would also be beneficial [32–34].

### A focus on career development

A recent study on faculty members’ participation in faculty development [35] indicated that the study participants believed that faculty development referred to their general development as faculty members. That is, they saw faculty development as the *development* of themselves as faculty members, including personal and career development, and not merely the enhancement of specific competencies related to teaching, research or administration. Interestingly, however, the literature does not report many faculty development programmes focusing on career development [36, 37], despite the fact that faculty members welcome the opportunity to identify career goals and values, develop collaborative relationships, and acquire skills to further their career path [36, 38]. Given that faculty members are our most important resource, it would seem that an investment in career development through faculty development represents a critical step forward. Programmes in this area could focus on academic identity formation, career planning (including an overview of different career paths) and the value of mentorship. In fact, mentorship can enhance recruitment, promote retention, and create an environment that enriches the academic role [39, 40], and as such, should be viewed as both a content area and a strategy in developing faculty. Time management, prevention of burnout, and promotion of well-being should also be considered as vital areas for faculty development.

In summary, medical teachers and educators need to be prepared for complex and demanding roles that include teaching, leadership and administration, research and scholarship [7], and career development, and faculty development initiatives should lead the way.

## Recognizing the role of faculty development in promoting curricular and organizational change

Faculty development can play an important role in promoting curricular and organizational change [2]. That is, it can help to promote teaching as a scholarly activity and create an educational climate that encourages and rewards educational leadership, innovation and excellence [41]. In addition, faculty development can help to build consensus, generate enthusiasm, and support curricular change [42, 43]. It can also contribute to changing the institutional culture by addressing the formal, informal, and hidden curriculum [44], and by enhancing organizational capacities [45]. For example, faculty development can promote culture change by helping to develop institutional policies that support and reward excellence in teaching, communicate the expectation of professionalism among all faculty members, encourage a re-examination of criteria for academic promotion if appropriate, and provide educational resources for junior and senior faculty members as needed. The latter might take the form of administrative support, timely provision of information (e.g., online educational resources), or new professional development opportunities. In our own setting, faculty development has played a valuable role in curricular change [43], the recognition of excellence in teaching [46], and the overall profile of teaching and learning.

Teachers in all settings face many competing demands and priorities. Faculty developers should work together with other educational leaders to help clarify expectations, protect time for teaching, and provide appropriate support for innovation and excellence. Swanwick [47] has stated that faculty development should be ‘an institution-wide pursuit with the intent of professionalizing the educational activities of teachers, enhancing educational infrastructure, and building educational capacity for the future.’ Working together across institutional portfolios to enhance the role of faculty development in producing organizational change would be strategically worthwhile.

## Incorporating notions of work-based learning and communities of practice into faculty development

The current faculty development literature primarily describes formal, structured activities, such as workshops and seminars, fellowships and other longitudinal programmes, and degree programmes, as the major method of delivery [2]. However, a number of recent articles have indicated the role of informal learning [48] and social factors [49] in faculty development as well as the value of faculty development in building communities of practice [50]. Looking forward, we should consider how faculty development can capitalize on the notions of work-based learning and communities of practice to promote the development of faculty members.

Work-based learning, which has been defined as learning *for* work, learning *at* work, and learning *from* work [47], is fundamental to the development of clinical and classroom teachers for whom ‘learning on the job’ is often the first entry into teaching. In fact, it is in the everyday workplace—where teachers conduct their clinical, research and educational activities—that learning most often takes place [7]. It would therefore be important for teachers and educators to see their everyday experiences as ‘learning experiences’ and to be encouraged to reflect with colleagues and students on what has occurred in the clinical or classroom setting. It would also be appropriate to bring faculty development to the workplace. Interestingly, faculty development activities have traditionally been conducted away from the teacher’s place of work, requiring participants to take their ‘lessons learned’ back to their own contexts. Perhaps it is time to reverse this trend and think about how we can enhance the learning that takes place in the work environment [51]. Peer coaching [52], which is sometimes called co-teaching or peer observation, can also complement work-based faculty development, as it enables individualized learning, increased collaboration and joint problem-solving.

The notion of a ‘community of practice’ is closely tied to that of work-based learning. Barab et al. [53] have defined a community of practice as a ‘persistent, sustaining, social network of individuals who share and develop an overlapping knowledge base, set of beliefs, values, history and experiences focused on a common practice and/or mutual enterprise’. As mentioned earlier, becoming a member of a teaching community can be a critical step in becoming a better teacher. Lave and Wenger [18] suggest that the success of a community of practice depends on five factors: the existence and sharing by the community of a common goal; the existence and use of knowledge to achieve that goal; the nature and importance of relationships formed among community members; the relationships between the community and those outside it; and the relationship between the work of the community and the value of the activity. A community also requires a shared repertoire of common resources, including language, stories, and practices [54]. In diverse ways, belonging to a community of practice builds on the collegiality that we often witness in clinical medicine and can be an important venue for faculty development, which in turn can lead to the development of a community of practice [50]. As leaders in medical education, we need to help our colleagues *value* the community of which they are a part (e.g., by celebrating its existence, members and resources) and *find* community (e.g., by building new networks, creating opportunities for exchange and support, and sustaining relationships) [7].

## Making faculty development an expectation for all faculty members

In recent years, regulatory bodies have started to pay attention to the accreditation of teachers and teaching [5, 6]; they have also highlighted the importance of faculty development in the certification of educators and the professionalization of medical education [55]. In the UK, for example, the role of teacher is increasingly recognized as a core professional activity that cannot be left to chance, aptitude, or inclination [56], and participation in staff development is becoming the norm. In North America, however, faculty development is a voluntary activity, and as some have said, ‘those who need faculty development the most attend the least’ [35]. As a consequence, many educators are now questioning whether faculty development should be made an expectation of all faculty members.

Interestingly, in 2008, the Association of Universities in the Netherlands stimulated the educational training and certification of all university teachers by affirming that all teachers must attain ‘basic qualifications’ in teaching [57]. Moreover, based on a national framework of teaching competencies [58], diverse programmes have developed across the country. The specific design of each programme is context-dependent, but all programmes address discipline-specific content and knowledge as well as the following topics: instructional design, instructional methods (e.g., lecturing, small group facilitation), assessment and evaluation, and generic skills (e.g., cooperation and teamwork) [59]. Portfolios are often used for assessing faculty progress in the attainment of basic teacher qualifications and some universities tailor the programmes to faculty members’ previous experiences (e.g., a course for senior educators). In many ways, it would be worthwhile for other countries to look at both the Dutch and UK experience to see if some of the ‘lessons learned’ might be pertinent to local contexts. We should also heed McLean et al.’s recommendations [60], as they suggest that faculty development should be integral to the mission of every medical school, that there should be formal preparation for anyone who teaches students, and that provision should be made for initial and ongoing professional development of all faculty members.

## Promoting scholarship to ensure that research informs practice

Research on the impact of faculty development activities has shown that overall satisfaction with programmes is high and that participants recommend these activities to their colleagues. Teachers also report a positive change in attitudes towards teaching as well as self-reported changes in knowledge about educational principles and specific teaching behaviors [22]. Other benefits include increased personal interest and enthusiasm, improved self-confidence, a greater sense of belonging to a community, and educational leadership and innovation [7].

However, despite numerous programme descriptions, there has been a paucity of research demonstrating the effectiveness of most faculty development activities [22, 41]; few programmes have conducted comprehensive evaluations and data to support the efficacy of these initiatives have been lacking. Of the studies that have been conducted in this area, many have relied on the assessment of participant satisfaction; some have evaluated the impact on learning or performance, while others have examined the long-term impact of specific interventions. More importantly, most of the research has relied on self-report rather than objective outcome measures or observations of change, and common problems have included a lack of control or comparison groups, heavy reliance on self-report measures of change, and small sample sizes [22]. There is clearly a need for more rigorous research designs and a greater use of qualitative and mixed methods to capture the complexity of faculty development interventions. The use of newer methods of performance-based assessment, incorporating diverse data sources, is also indicated, as is the value of process-oriented studies comparing different faculty development strategies and the maintenance of change over time [7].

In a recent article, O’Sullivan and Irby [61] outlined an agenda for research in faculty development. They wisely suggest that we need to expand our conceptualization of faculty development and examine educational processes and outcomes on two communities of practice: the community created among participants in faculty development programmes and the communities of teaching practice in the workplace (where teaching occurs). As they state, ‘for the faculty development community, the key components are the participants, programme, content, facilitator, and context in which the programme occurs’ [61]. For the workplace community, associated components include ‘relationships and networks of association in that environment, the organization and culture of the setting, the teaching tasks and activities, and the mentoring available to that community’ [61]. The model proposed by O’Sullivan and Irby informs a new set of research questions that aligns closely with the notions of work-based learning and communities of practice described earlier. Irrespective of the model adopted, however, it behoves us to systematically evaluate the work done in this field and carry out studies that will advance our understanding of how faculty members develop as they pursue their individual career trajectories.

## Aiming for 6/6 by 2020

Normal visual acuity is expressed as 6/6 in Europe and 20/20 in North America. It is hoped that, collectively, we will be able to reach this level of acuity in faculty development. To accomplish this objective, this perspective on faculty development highlights a number of areas where change might be indicated, depending on local contexts and needs. To pave the way, a review of Kotter’s steps for ‘leading change’ might also be helpful [62]. These steps include: establishing a sense of urgency; forming a guiding coalition; creating a vision; communicating the vision; empowering others to act on the vision; generating short-term wins; consolidating gains and producing more change; and anchoring the change in the culture. As faculty developers, we should ask ourselves why a particular change is needed, and if it is, we should work together with colleagues to create and communicate our vision, promote buy-in, identify opportunities and threats, create short-term wins, and anchor the change in the culture before pursuing a new direction.

In considering the changing landscape of faculty development, it is also interesting to observe that a number of the recommendations included in this article do not differ from suggestions made in 2000 [41]. For example, why have faculty development initiatives continued to focus primarily on the educational development of faculty members? Similarly, why have we not been able to conduct more systematic research in this domain? In my opinion, these questions raise interesting avenues for future research. They also suggest that we need to carefully consider the social and cultural contexts in which faculty development initiatives unfold. Faculty development offerings are often designed in response to ‘urgent’ educational needs, and ‘service’ to the community is frequently the first priority. This observation might help to explain the emphasis on teaching improvement and the apparent focus on the individual. At the same time, funding for research in this field is often limited, and as a result, programme evaluation may take precedence over more carefully designed research studies. However, with a significant increase in the number of individuals trained to conduct research in medical education, and a concomitant rise in centres dedicated to medical education research (which are the norm in most medical schools in the Netherlands), a more focused research programme, and increased scholarly activity in this area, may be timely. In addition, collaboration across sites and institutions, as well as the development of networks to foster and support scholarship, would help to move this strategic priority forward.

As stated in 2000, ‘the changing roles of faculty members will continue to drive the changing nature of faculty development practices, as will the evolution of the organizations in which we work’ [41]. We must also remember to think about faculty development beyond our local contexts [2] and be prepared to collaborate with partners around the world, sharing our expertise, accumulated ‘know how’ and resources. Swanwick [47] outlined three drivers for faculty development in postgraduate medical education: increasing accountability, the pursuit of excellence, and the professionalization of medical education. These drivers are equally important in this context, as we look forward to pursuing innovation and excellence in health professions education with the ultimate goal of improving health care delivery and practice.

## Essentials

- Faculty development has a key role to play in individual and organizational change.
- Faculty development initiatives should address faculty members’ multiple roles.
- Faculty development activities should be grounded in a theoretical framework.
- Faculty development programs should include work-based learning and communities of practice.
- Faculty development practices should be systematically assessed and informed by research findings.
